# Prevalence and influencing factors of malocclusion in adolescents in Shanghai, China

**DOI:** 10.1186/s12903-023-03187-5

**Published:** 2023-08-24

**Authors:** Jiaming Yin, Hao Zhang, Xiaoli Zeng, Jin Yu, Huning Wang, Yiwei Jiang, Dongxin Da, Qiang Li, Ying Zhang

**Affiliations:** 1 Department of Preventive Dentistry, Shanghai Stomatological Hospital, Fudan University, 356 East Beijing Rd, Shanghai, 200001 China; 2 Shanghai Key Laboratory of Craniomaxillofacial Development and Diseases, Fudan University, Shanghai, China; 3 Department of Orthodontics, Shanghai Stomatological Hospital, Fudan University, 356 East Beijing Rd, Shanghai, 200001 China

**Keywords:** Malocclusion, Prevalence, Teenagers, Permanent dentition, Risk factors

## Abstract

**Background:**

The main purpose of the study was to investigate the prevalence and related risk factors of malocclusion in permanent dentition among adolescents in Shanghai, and provide basic data for government’s preventive strategies and intervention plans.

**Methods:**

1799 adolescents aged 11–15 years old from 18 middle schools in 6 districts of Shanghai were recruited to investigate oral health status and related risk factors using cluster random sampling method in 2021. Malocclusion and caries were examined by on-site inspection. The investigation criteria referred to Bjoerk and the recommendation of the WHO. The malocclusion inspection items included molars relationship, canine relationship, overbite, overjet, midline displacement, anterior crossbite, posterior crossbite, scissors bite, crowding and spacing. The subjects were asked to fill in a questionnaire including parents’ education level, oral health behaviors and dietary habits. The chi-square test and logistic regression analysis were used to analyze the relationship between malocclusion and risk factors.

**Results:**

1799 adolescents were included in the study and the prevalence of malocclusion in adolescents in Shanghai was 83.5%, and the proportion of molar relationship class I, class II, and class III was 48.9%, 14.7%, and 19.0%, respectively. The most common occlusal characteristic of malocclusion was anterior crowding, followed by midline irregularities and deep overbite, with prevalence rates of 44.8%, 39.0% and 38.6%, respectively. The prevalence rate of adolescents with caries was 34.3%. Those who had dental caries and preferred soft food were more likely to have abnormal occlusal characteristics (p < 0.05).

**Conclusion:**

The prevalence of malocclusion in adolescents in Shanghai is high, so it is of great significance to strengthen oral health education, allocate proper preventive strategies and carry out the early correction if necessary.

## Background

Malocclusion refers to deformities of teeth, jaws, and faces arising from congenital genetic and acquired environmental factors. Patients mainly show individual tooth dislocation, abnormal dental archmorphology, and abnormal tooth arrangement [1]. It is a kind of common oral disease, which is defined by the World Health Organization as the third most common oral diseases bisides dental caries and periodontal disease [2]. Malocclusion not only affects the dental and maxillofacial function, maxillofacial development and facial esthetics, but also has a negative influence over social, emotional and functional aspects [3]. There are many reasons for malocclusion, mainly including genetic factors and environmental factors, which includes congenital maternal factors during pregnancy, fetal development factors and acquired developmental factors and lousy behavior habits. Permanent dentition generally refers that all the deciduous teeth in the dental arch are replaced by permanent teeth, and the dentition has a stable bite. According to the current literature, the prevalence of malocclusion in permanent dentition varied considerably in different countries and regions. For example, Sidlauskas [4] reported that the prevalence of malocclusion in students aged 7–15 in Lithuania was 84.6%. Thilander [5] reported that the prevalence of malocclusion in Sweden was 73.8%, and Abu [6] reported the prevalence rate in 13-15-year-old North Jordanian schoolchildren in the permanent dentition was 92% referring to Bjork registration method. In China, the last large-scale epidemiological survey was the nationwide survey on the prevalence of malocclusion in adolescents organized by Professor Fu in 2000. It was found that the prevalence of malocclusion in Chinese adolescents in permanent dentition was 72.97% referring to Angle’s classification [7]. In addition, other investigations were conducted in various provinces in China, and the results of investigations in various provinces and regions showed that the incidence of malocclusion was high and quite various in each region [8]. Therefore, only if we realize the prevalence and influencing factors of malocclusion in our country and formulate effective intervention measures, can we reduce the occurrence of oral diseases effectively. In Shanghai, one of the most economically developed cities in China, epidemiological investigation on malocclusion of deciduous dentition and mixed dentition was carried out a few years ago. Yu [9]’s investigation in 2017 found that prevalence of malocclusion in the mixed dentition in children aged 7–9 years was 79.4%and Zhou [10] found that the prevalence of malocclusion in the primary dentition in children aged 3–5 years from kindergartens was 83.9% in 2016 referring to Bjork registration method. However, there was little investigations on malocclusion of permanent dentition. So the primary purposes of the cross-sectional study were to explore the condition and related risk factors of malocclusion in the permanent dentition of adolescents in Shanghai and provide essential data for government’s preventive strategies and early intervention plans of malocclusion.

## Methods

### Research subjects

A cross-sectional study was carried out in 2021 in shanghai. In order to ensure the representativeness of samples, the cluster random sampling method was adopted, and 6 districts in Shanghai (Putuo District, Hongkou District, Minhang District, Pudong District, Xuhui Districtand Jing ‘an District) were randomly selected. Three middle schools were randomly selected from each district, and 140 teenagers aged 11–15 were randomly selected from each middle school.Finally 1799 adolescents were included in the study, including 955 boys and 844 girls. The study was reviewed and approved by the Medical Ethics Committee of China Oral Health Foundation(2021-001).

### Inclusion and exclusion criteria

The inclusion criteria were as follows: 1.aged between 11 and 15 years old; 2. had no physical disability; 3. in permanent dentition. The students with the following characteristics were excluded from the study: 1.orthodontic treatment history; 2.uncooperative; 3.who had systemic diseases. All the subjects were informed of the purpose and process of the epidemiological investigation and signed informed consent.

### Data collection

#### Clinical examination

The children lay on the portable dental chair and were examined by dentists, who used CPI probe and stomatoscope under the light of a headlamp. The investigation criteria refer to Bjoerk [11], the inspection items included molars relationship, canine relationship, overbite, overjet, midline displacement, anterior crossbite, posterior crossbite, scissors bite, crowding and spacing. Malocclusion was diagnosed when the subjects met any of the following indicators:1.anterior crossbite; 2.deep overbite:coverage of the mandibular incisors by the maxillary incisor > 1/3;3.increased overjet:distance of the mesial corner of maxillary incisor to the corresponding mandibular incisor > 3 mm; 4.posterior crossbite or scissors bite; 5.anterior spacing or crowding; 6.midline displacement; 7.molar Class II or Class III. Dental caries was measured by the decayed, missing, filled teeth (DMFT) score by recording the number of teeth that were decayed, missing due to caries and filled according to the recommendation of the WHO [2]. Those with DMFT score > 0 were considered as having dental caries experience.

#### Questionnaire examination

The questionnaires were completed by students under the investigators’ guidance which contained questions about their parents’ education level, children’s oral behaviours and dietary habits.

### Inspection items

1. Sagittal anomalies.
  1.1 Permanent molars relationship.
    Class I, the mesiobuccal cusp of the maxillary first permanent molar occluded with the mesiobuccal groove of the mandibular first permanent molar (normal relation), or up to or equal to 1/2 cusp width post-normal or prenormal relation; Class II (distal), more than 1/2 cusp width post-normal relation; Class III (mesial), more than 1/2 cusp width pre-normal relation. The molar relationship was recorded as class II or class III, if it was class I on one side and class II or class III on the other. Children with class II molar relation on one side and class III on the other side were recorded as mixed.
  1.2 Permanent canine relationship.
    Equal to Angel’s classification. The canine relationship was recorded as class II or class III, if it was class I on one side and class II or class III on the other. Children with class II canine relation on one side and class III on the other side were recorded as mixed.
  1.3 Overjet.
    Distance of the palatal surface of the mesial corner of the most protruded maxillary incisor to the labial surface of the corresponding mandibular incisor.(>0 mm, ≤ 3 mm, normal; >3 mm, ≤ 5 mm, mild; >5 mm, ≤ 8 mm, moderate; >8 mm, severe).
  1.4 Anterior crossbite.
    One or more of the maxillary incisors occluded lingually to the mandibular incisors.

2. Vertical anomalies.
  2.1 overbite.
    Coverage of the mandibular incisors by the most protruded fully erupted maxillary incisor.(>0, ≤ 1/3, normal; >1/3, ≤ 1/2, mild; >1/2, ≤ 2/3, moderate; >2/3, severe > 2/3).

3. Transversal anomalies.
  3.1 Midline displacement.
    Mandibular midline deviated 2 mm or more to the maxillary midline.
  3.2 Posterior crossbite.
    one or more of the maxillary primary molars occluded the lingual to the buccal cusps of the corresponding mandibular teeth.
  3.3 Scissors bite.
    one or more maxillary primary molars occluded the buccal to the buccal surfaces or the lingual to the lingual surfaces of the corresponding mandibular teeth.

4. Space discrepancies.
  4.1 Crowding (anterior, posterior; maxillary, mandibular) > 2 mm.
  4.2 Spacing (maxillary, mandibular) > 2 mm.

5. Caries examination.
  Caries was determined according to the criteria of the fifth edition of Oral Health Survey Method [2] issued by the National Health and Family Planning Commission. Caries was determined when the subjects met any of the following indicators: (I) there were obvious cavities or enamel damage in the gap or smooth surface of teeth; (II) there were obvious lesions that could be detected to soften the bottom or wall of the cave; (III) there was a filling in the tooth; (IV) the tooth had been filled with caries; (V) lack of caries.

### The quality control

The examiners were experienced stomatologists who have been engaged in dental practice for more than 5 years. They were proficient in the examination of malocclusion. In order to avoid the bias of the examiners, orthodontic experts carried out special training for malocclusion examination and unified the standard before the on-site investigation. Moreover, orthodontic specialists’ examinations was used as reference to test the standard consistency of the index of “crowding”. Finally, the kappa values were all greater than 0.8, suggesting that the reliability of the results is better.

### Statistical analysis

Epidata 3. 0 software (The EpiData Association, Odense, Denmark) was used to establish a database after data collection.All data were processed by SPSS26.0 software (IBM Corporation, Armonk, NY, USA) for analysis. The rates of malocclusion and occlusal characteristics were reported by gender. The rates difference between girls and boys was tested with chi-squared test. To identify risk factors associated with malocclusion, we used chi-squared test to compare the rates of malocclusion between groups with different characteristics, such as different parents’ education level, oral health behaviors and dietary habits. And then the related factors were included into a stepwise binary logistic regression. The variables in the logistic model significantly positively with malocclusion were considered as independent risk factor. In all analysis, statistical significance was assumed when p < 0.05.

## Results

1799 adolescents were included in the study, including 955 boys and 844 girls. And we noticed that 570 adolescents who had a history of orthodontic treatment or were under treatment were excluded. We found the prevalence of malocclusion in permanent dentition was 83.5%(Table 1). The prevalence of malocclusion in boys and girls was 84.4% and 82.5%, respectively. And there was no significant difference between boys and girls (p > 0.05).

**Table 1:** Prevalence of malocclusion in adolescents in Shanghai

	Normal occlusion		Malocclusion		*P*
Gender					0.274
Boys	148	15.6	803	84.4	
Girls	146	17.5	689	82.5	
Total	294	16.5	1492	83.5	

The composition and prevalence of occlusal characteristic in adolescents in Shanghai are in Table 2. Among the various occlusal characteristics of malocclusion, the prevalence of anterior crowding (44.8%) was the highest, followed by midline displacement (39.0%), deep overbite (38.6%) and increased overjet (32.0%). The prevalence of anterior crowding was the highest in boys (43.4%), followed by deep overbite (42.3%), midline displacement (36.1%) and increased overjet (35.2%). The prevalence of anterior crowding was the highest in girls (46.3%), followed by midline displacement (42.2%), deep overbite (34.3%) and increased overjet (28.3%).

**Table 2:** Composition and prevalence of occlusal characteristic in adolescents

occlusal characteristic	Boys		Girls		Total			*P*
	n	%	n	%	n	%		
increased overjet	336	35.2	238	28.3	574	32.0	9.841	0.002
deep overbite	404	42.3	288	34.3	692	38.6	12.128	0
midline displacement	345	36.1	355	42.2	700	39.0	6.964	0.008
posterior crossbite	50	5.2	53	6.3	103	5.7	0.942	0.332
scissors bite	50	5.2	43	5.1	93	5.2	0.011	0.917
anterior crossbite	103	10.8	78	9.3	181	10.1	1.126	0.289
Anterior crowding	414	43.4	389	46.3	803	44.8	1.533	0.216
Anterior spacing	110	11.5	73	8.7	183	10.2	3.863	0.049

The general prevalence of malocclusion is presented in Tables 3 and 4. The most common type of molars relationship in adolescents was the Class I relationship (65.5%), followed by Class III relationship (18.9%) and Class II relationship (14.7%). The relationship of the canine was recorded. 68.4% of children showed a Class I canine relationship, 16.3% of children were Class II, and 14.6% of them were Class III. The probability of the increased overjet was 32% and overjet (3,5 mm) accounted for the majority. The prevalence of deep overbite was 38.6% and overbite(3,5 mm) accounted for the majority.

**Table 3:** Composition and prevalence of sagittal occlusal characteristic

Sagittal characteristic	Gender				*P*	Total	
	Boys		Girls				
	n	%	n	%		n	%
molars relationship					0.003		
Class I	589	61.7	588	70.0		1177	65.6
Class II	157	16.4	106	12.6		263	14.7
Class III	199	20.8	141	16.8		340	18.9
Mixed	10	1.0	5	0.6		15	0.8
canine relationship					0.039		
Class I	625	65.4	603	71.7		1228	68.4
Class II	168	17.6	125	14.9		293	16.3
Class III	155	16.2	107	12.7		262	14.6
Mixed	7	0.7	6	0.7		13	0.7
overjet					0.008		
≤ 3 mm	618	64.8	603	71.7		1221	68.0
3−5 mm	214	22.4	164	19.5		378	21.1
5−8 mm	90	9.4	57	6.8		147	8.2
≥ 8 mm	32	3.4	17	2.0		49	2.7
anterior crossbite					0.289		
yes	103	10.8	78	9.3		181	10.1
no	852	89.2	763	90.7		1615	89.9

Notes:students with Class II molar relation on one side and Class III on the other side was defined as mixed

**Table 4:** Composition and prevalence of vertical and transversal occlusal characteristic

Vertical and transversal characteristic	Gender				*P*	Total	
	Boys		Girls				
	n	%	n	%		n	%
overbite					0.001		
≤ 1/3	551	57.7	552	65.7		1103	61.4
1/3−1/2	223	23.4	179	21.3		402	22.4
1/2–2/3	114	11.9	75	8.9		189	10.5
≥ 2/3	67	7.0	34	4.0		101	5.6
posterior crossbite					0.332		
yes	50	5.2	53	6.3		103	5.7
no	904	94.8	787	93.7		1691	94.3
scissors bite					0.917		
yes	50	5.2	43	5.1		93	5.2
no	904	94.8	795	94.9		1699	94.8
midline displacement					0.008		
yes	345	36.1	355	42.2		700	39.0
no	610	63.9	486	57.8		1096	61.0

Table 5 indicated the prevalence of anterior teeth crowding was 44.8%, the prevalence of anterior crowding of the maxillary or mandibular teeth were 11.9% and 20.2%, respectively. 20.2% of students had both upper and lower teeth crowding. The prevalence of anterior teeth spacing was 44.8%, the rate of anterior crowding of the maxillary or mandibular teeth was 4.3% and 3%, respectively. 2.9% of the students had spacing of both upper and lower teeth.

**Table 5:** other occlusal characteristics

Space discrepancies	Gender				*P*	Total	
	Boys		Girls				
	n	%	n	%		n	%
crowing					0.262		
Maxillary	100	10.5	114	13.6		214	11.9
Mandibular	114	11.9	103	12.3		217	12.1
both	194	20.3	168	20.0		362	20.2
spacing					0.017		
Maxillary	55	5.8	22	2.6		77	4.3
Mandibular	25	2.6	28	3.3		53	3.0
both	29	3.0	23	2.7		52	2.9

We found the number of adolescents with caries was 617, the caries prevalence rate was 34.3%. The prevalence of malocclusion in those with caries was 86.4%. The prevalence of malocclusion in adolescents with caries was 1.396 times that in adolescents without caries. At the same time,the prevalence of malocclusion in adolescents who preferred soft food was 1.444 times that in adolescents without caries who preferred hard food (Table 6). Those who had dental caries and preferred soft food were more likely to have abnormal occlusal characteristics (p < 0.05).

**Table 6:** Binary logistic analysis of malocclusion and related factors

	*P*	OR	95%CI
Dietary			
hard			1
soft	0.005	1.444	1.115–1.871
Caries			
no			1
yes	0.030	1.364	1.030–1.805

## Discussion

At present, there is no generally accepted criteria to define normality or abnormality as regards occlusal status. Numerous studies used different criteria including the Index of Orthodontic Treatment Need (IOTN) [11], the Dental Aesthetic Index (DAI) [12], the Angle classification method. However, none of these methods could be simultaneously systematic. So other authors preferred disaggregated measures of the major occlusal characteristics, with the epidemiological registration of malocclusion developed by Bjork [13]. The prevalence of malocclusion varied from 32.5 to 87% [14–18] among different investigations in the different countries and regions. Many factors contributed to it, including ethnic differences, regional economic differences, dietary habits, the geographic area, the time of the study, sampling methods, sample size and the criteria used for the survey. Our survey adopted Bjork registration method and found the total prevalence rate of malocclusion in the adolescents in Shanghai was 83.5%, which was higher than 72.97% in the early permanent dentition investigated by Fu [7] in 2000. Over time, the rate of malocclusion generally has increased. A number of factors contributed to this, including the increasing prevalence of dental caries and the increasingly refined diet,etc.

Our study found that malocclusion was well associated with dental caries and dietary habits. With the development of our country’s economy, students’ diets has changed more refined. Kiliaridis [19]’findings indicated that dietary pattern had a significant effect on chewing function and the masticatory muscle function influenced not only bone remodeling in local areas due to direct muscle action, but also the general craniofacial growth pattern. Fine dietary patterns caused that their facial muscles and jaw bones were not stimulated enough during development. In addition, Our investigation and Anand’s [20]study both found that soft diet led to increased malocclusion.

We found the dental caries prevalence rate in adolescents was 34.3%. Caries of permanent teeth were often accompanied by caries of deciduous teeth, while deciduous tooth caries could cause tooth defects, premature loss of deciduous teeth and inadequate chewing function, which could lead to the occurrence of malocclusion. Dental caries led to the loss of hard tissue in healthy teeth, resulting in the reduction of arch length and crowding of dentition. There was a positive correlation between malocclusion and dental caries among adolescents. So the prevention and treatment of dental caries was of great significance for the prevention of malocclusion.

In our survey, we found that the highest prevalence of malocclusion in adolescents was anterior crowding with a rate of 44.8%, much higher than the 14.3% reported by Katalin [21] in Hungary, but lower than the 47.5% reported by Bianca [22] in Romania. The proportion of crowding in the maxillary arch was 11.9%, and that in the mandibular arch was 12.1%, which was similar to the 13.9% of maxillary arch and 12.2% of mandibular arch in Libyan adolescents reported by Bugaighis [23]. Females were more likely than males to have anterior tooth congestion, with the prevalence rate being 43.4% for boys and 46.3% for girls. This was consistent with Thilander [24] ‘s investigation in Colombia. If there is crowding in the mixed dentition, the likelihood of crowding in anterior teeth will increase in the permanent dentition. Yu [9] found that the prevalence of anterior crowding of the mixed dentition in Shanghai was as up to 28.4%, and the high prevalence of the mixed dentition was also an important reason for the crowding of permanent dentition in the later period. There was a consensus that premature extraction of deciduous teeth might result in unfavorable drifts of deciduous or permanent teeth or both dentitions. This finally resulted in space loss in the developing dentition and decreased the arch length required for permanent teeth, exacerbating the arch’s crowding [25].

The proportion of midline displacement in the survey was 39%, lower than the 43.33% reported by Bianca [22] in Romania, 54% reported by Ciuffolo [26] in Italy, and 6.8% reported by Sundareswaran [27] in India. The most common reason of dental midline shift was early shedding of the deciduous teeth, which could lead to space and midline displacement in the permanent dentition.

The survey found that class II malocclusion of permanent dentition among adolescents in Shanghai accounted for 14.7%, which was lower than 25.4% in Libya [23] and 38.2% reported among 12 to 15-year-old adolescents in Japan. The incidence of class III malocclusion was 18.9%, which was higher than 3.7% in Libya [23], 4.3% in Italy [28], and 7.8% in Iran [29]. Warren [30] found sustained pacifier habits in infancy, including those of 24 to 47 months, were associated with Class II molar relationships. There were few investigations on the canine relationship, so we paid special attention to this canine relationship. Among adolescents in Shanghai, canine II accounted for 16.3% and canine III accounted for 14.6%. Silva [31] found those with class II canines had a 56% higher prevalence of severe trauma than those with class I and III canines. And Rai [32] revealed significant association of digit sucking habit and pacifier sucking habit with class II canine relationship.

We found that there were 570 students who were currently undergoing or previously underwent orthodontic treatment, accounting for 22.6% of the total sample number. It was higher than the 2.63% proportion of adolescents aged 12–14 years receiving orthodontic treatment in Jiangxi province reported by Xu [18] in 2019 and 21% of adolescents aged 11–14 years in Italy reported by Perillo [29] in 2009. But it was lower than the 28% of adolescents aged 12–13 years reported by Josefsson [33] in Sweden in 2005. The rate of orthodontic treatment was related to the economic level and parents’ orthodontic awareness in various countries and regions. In Shanghai, which is one of the most economically developed cities in China, parents had better oral health awareness and tend to pay more attention to their children’s oral health. And the high prevalence of malocclusion also contributed to the high demand for correction. At the same time, we excluded these children who had a history of orthodontic treatment or were currently undergoing orthodontic treatment, because we had no way to know their occlusal features before treatment, and their current features had been artificially intervened. In fact, their previous occlusal characteristics were almost anomalous. We speculated that the previous exclusion introduced some representativeness bias. If we included these adolescents who had received orthodontic treatment, the prevalence of malocclusion would be 87.5%. The rate was roughly accurate but better research methods ought to be devised to make the data more precise.

There has been controversy over whether there was difference in the prevalence of malocclusion between boys and girls. Shen Lu [34] found there was no significant difference in malocclusion by gender through a piece of meta-analysis. This study found that there was no significant difference in the prevalence of malocclusion between genders, but there were statistical differences in the specific occlusal features of deep overbite irregular midline. Therefore, further research should be used to explore whether gender is an influential factor in malocclusion.

## Conclusions

Our oral epidemiological study showed the prevalence of Malocclusion in adolescents in shanghai was relatively high. Therefore, it was of great significance for government to strengthen oral health education and allocate proper preventive strategies. Teenagers and their parents should pay more attention to malocclusion and check their teeth regularly, actively treat dental caries and carry out early intervention if necessary.

## Data Availability

The datasets used and analyzed during the current study are available from the corresponding author on reasonable request.
